# Blood biomarkers confirm subjective cognitive decline (SCD) as a distinct molecular and clinical stage within the NIA-AA framework of Alzheimer´s disease

**DOI:** 10.1038/s41380-025-03021-0

**Published:** 2025-04-18

**Authors:** David Mengel, Ester Soter, Julia Maren Ott, Madeleine Wacker, Alejandra Leyva, Oliver Peters, Julian Hellmann-Regen, Luisa-Sophie Schneider, Xiao Wang, Josef Priller, Eike Spruth, Slawek Altenstein, Anja Schneider, Klaus Fliessbach, Jens Wiltfang, Niels Hansen, Ayda Rostamzadeh, Emra Düzel, Wenzel Glanz, Enise I. Incesoy, Katharina Buerger, Daniel Janowitz, Michael Ewers, Robert Perneczky, Boris Rauchmann, Stefan Teipel, Ingo Kilimann, Christoph Laske, Sebastian Sodenkamp, Annika Spottke, Johanna Brustkern, Frederic Brosseron, Michael Wagner, Melina Stark, Luca Kleineidam, Kai Shao, Falk Lüsebrink, Renat Yakupov, Matthias Schmid, Stefan Hetzer, Peter Dechent, Klaus Scheffler, David Berron, Frank Jessen, Matthis Synofzik, the DELCODE study group, Oliver Peters, Josef Priller, Eike Spruth, Slawek Altenstein, Anja Schneider, Klaus Fliessbach, Jens Wiltfang, Niels Hansen, Ayda Rostamzadeh, Emra Düzel, Wenzel Glanz, Enise I. Incesoy, Katharina Buerger, Daniel Janowitz, Robert Perneczky, Stefan Teipel, Ingo Kilimann, Christoph Laske, Sebastian Sodenkamp, Annika Spottke, Frederic Brosseron, Michael Wagner, Melina Stark, Renat Yakupov, Peter Dechent, Frank Jessen

**Affiliations:** 1 Division Translational Genomics of Neurodegenerative Diseases, Hertie-Institute for Clinical Brain Research and Center of Neurology, University of Tübingen, Hoppe-Seyler-Str. 3, 72076 Tübingen, Germany; 2 German Center for Neurodegenerative Diseases (DZNE), University of Tübingen, Otfried-Müller-Straße 27, 72076 Tübingen, Germany; 3 German Center for Neurodegenerative Diseases (DZNE), Robert-Rössle-Straße 10, 13125 Berlin, Germany; 4 Charité – Universitätsmedizin Berlin, corporate member of Freie Universität Berlin and Humboldt-Universität zu Berlin, Institute of Psychiatry and Psychotherapy, Charitéplatz 1, 10117 Berlin, Germany; 5 Charité – Universitätsmedizin Berlin, Department of Psychiatry and Neurosciences, corporate member of Freie Universität Berlin and Humboldt-Universität zu Berlin, Charitéplatz 1, 10117 Berlin, Germany; 6 German Center for Mental Health (DZPG), partner site Berlin, Charitéplatz 1, 10117 Berlin, Germany; 7 Charité – Universitätsmedizin Berlin, Experimental and Clinical Research Center (ECRC), Lindenberger Weg 80, 13125 Berlin, Germany; 8 Department of Psychiatry and Psychotherapy, Charité, Charitéplatz 1, 10117 Berlin, Germany; 9 School of Medicine, Technical University of Munich, Department of Psychiatry and Psychotherapy, Ismaninger Str. 22, 81675 Munich, Germany; 10 University of Edinburgh and UK Dementia Research Institute (UK DRI), Chancellor’s Building, 49 Little France Crescent, Edinburgh, EH16 4SB UK; 11 German Center for Neurodegenerative Diseases (DZNE), Sigmund-Freud-Straße 27, 53127 Bonn, Germany; 12 Department for Cognitive Disorders and Old Age Psychiatry, University Hospital Bonn, Sigmund-Freud-Straße 25, 53127 Bonn, Germany; 13 German Center for Neurodegenerative Diseases (DZNE), Von-Siebold-Straße 3a, 37075 Göttingen, Germany; 14 Department of Psychiatry and Psychotherapy, University Medical Center Göttingen, University of Göttingen, Von-Siebold-Straße 3a, 37075 Göttingen, Germany; 15 Neurosciences and Signaling Group, Institute of Biomedicine (iBiMED), Department of Medical Sciences, University of Aveiro, Campus Universitário de Santiago, 3810-193 Aveiro, Portugal; 16 Department of Psychiatry, Faculty of Medicine and University Hospital Cologne, University of Cologne, Kerpener Str. 62, 50937 Cologne, Germany; 17 German Center for Neurodegenerative Diseases (DZNE), Leipziger Str. 44, 39120 Magdeburg, Germany; 18 Institute of Cognitive Neurology and Dementia Research (IKND), Otto-von-Guericke University, Leipziger Str. 44, 39120 Magdeburg, Germany; 19 Department of Psychiatry and Psychotherapy, University Clinic Magdeburg, Leipziger Str. 44, 39120 Magdeburg, Germany; 20 German Center for Neurodegenerative Diseases (DZNE), Feodor-Lynen-Str. 17, 81377 Munich, Germany; 21 Institute for Stroke and Dementia Research (ISD), University Hospital, LMU, Feodor-Lynen-Str. 17, 81377 Munich, Germany; 22 Department of Psychiatry and Psychotherapy, University Hospital, LMU, Nussbaumstraße 7, 80336 Munich, Germany; 23 Munich Cluster for Systems Neurology (SyNergy), Feodor-Lynen-Str. 17, 81377 Munich, Germany; 24 Ageing Epidemiology Research Unit (AGE), School of Public Health, Imperial College London, St. Mary’s Campus, Norfolk Place, London, W2 1PG UK; 25 Sheffield Institute for Translational Neuroscience (SITraN), University of Sheffield, 385a Glossop Road, Sheffield, S10 2HQ UK; 26 Department of Neuroradiology, University Hospital LMU, Marchioninistraße 15, 81377 Munich, Germany; 27 German Center for Neurodegenerative Diseases (DZNE), Gehlsheimer Straße 20, 18147 Rostock, Germany; 28 Department of Psychosomatic Medicine, Rostock University Medical Center, Gehlsheimer Straße 20, 18147 Rostock, Germany; 29 Section for Dementia Research, Hertie Institute for Clinical Brain Research and Department of Psychiatry and Psychotherapy, University of Tübingen, Hoppe-Seyler-Str. 3, 72076 Tübingen, Germany; 30 Department of Psychiatry and Psychotherapy, University of Tübingen, Calwerstraße 14, 72076 Tübingen, Germany; 31 Department of Neurology, University of Bonn, Sigmund-Freud-Straße 25, 53127 Bonn, Germany; 32 Department of Old Age Psychiatry and Cognitive Disorders, University Hospital Bonn, Sigmund-Freud-Straße 25, 53127 Bonn, Germany; 33 Department of Neurology, XuanWu Hospital of Capital Medical University, No. 45 Changchun Street, Xicheng District, Beijing, 100053 China; 34 Institute for Medical Biometry, University Hospital Bonn, Sigmund-Freud-Straße 25, 53127 Bonn, Germany; 35 Berlin Center for Advanced Neuroimaging, Charité – Universitätsmedizin Berlin, Philippstraße 13, 10115 Berlin, Germany; 36 MR-Research in Neurosciences, Department of Cognitive Neurology, Georg-August-University Göttingen, Von-Siebold-Straße 3a, 37075 Göttingen, Germany; 37 Department for Biomedical Magnetic Resonance, University of Tübingen, Hoppe-Seyler-Str. 3, 72076 Tübingen, Germany; 38 Clinical Memory Research Unit, Department of Clinical Sciences Malmö, Lund University, Sölvegatan 17, 223 62 Lund, Sweden; 39 Excellence Cluster on Cellular Stress Responses in Aging-Associated Diseases (CECAD), University of Cologne, Joseph-Stelzmann-Straße 26, 50931 Cologne, Germany

**Keywords:** Predictive markers, Neuroscience

## Abstract

Subjective cognitive decline (SCD) is proposed as an indicator of transitional disease stage 2 in the Alzheimer’s disease (AD) continuum. However, molecular and particularly longitudinal fluid biomarker data for this stage are still limited. This study aimed to determine whether blood-based biomarkers in amyloid-positive individuals with SCD (A + SCD) support the notion of stage 2 as a distinct stage between stages 1 and 3 of AD and to identify those at high risk for clinical progression. In a prospective multicenter study (DELCODE) involving 457 participants across the AD continuum, we analyzed plasma phospho-tau 181 (p181) and neurofilament light chain (NfL) and assessed their association with longitudinal cognition, hippocampal atrophy, and AD clinical stage transition. The results showed that baseline plasma p181 levels were elevated and increased more rapidly in A + SCD individuals compared to amyloid-positive cognitively unimpaired (A + CU) individuals (stage 1). NfL levels rose across A + CU, A + SCD, and amyloid-positive mild cognitive impairment (A + MCI, stage 3). In A + SCD, but not in A + CU, higher p181 levels predicted cognitive decline (PACC5) and transition to MCI. In conclusion, plasma p181 provides molecular biomarker evidence supporting A + SCD as a pre-dementia AD stage (stage 2) distinct from A + CU (stage 1) and helps identify individuals at risk for cognitive decline early in the AD continuum.

## Background

The pathophysiological processes of Alzheimer’s disease (AD) including amyloid and tau deposition unfold many years prior to the appearance of initial symptoms and subsequent progression to dementia [1]. This prolonged pre-dementia period presents a crucial window for potential interventions to be introduced, aiming to alleviate or defer the onset of cognitive decline [2–5]. The National Institute on Aging and Alzheimer’s Association (NIA-AA) working group’s updated research criteria delineated 6 progressive clinical stages evident in individuals along the AD continuum, which is identified by the presence of amyloid pathology with or without tau pathology [6]. Within the pre-dementia stages, this staging system includes a transitional stage 2 positioned between the fully asymptomatic stage 1 and stage 3 defined by mild cognitive impairment (MCI). A symptom that has been associated with stage 2 is subjective cognitive decline (SCD) [6, 7]. SCD is defined as self-experienced decline in cognitive functioning, while - in contrast to MCI - the performance on diagnostic neuropsychological tests is normal. In elderly individuals, SCD is associated with an increased risk of future cognitive decline [8], and may occur more than 15 years before dementia onset [9, 10]. Meta-analyses reported a conversion rate from SCD to MCI and dementia in 27% and 6–14%, respectively [11, 12]. Recent clinical research findings indicate that individuals experiencing subjective cognitive decline in the presence of amyloid pathology (A + SCD) show reduced cognitive and functional performance and face a significantly increased risk of progressing to MCI and dementia when compared to SCD individuals without AD pathology [13, 14], providing evidence that A + SCD might present a distinct pre-dementia AD disease stratum. However, there is a scarcity of comprehensive characterizations of molecular changes in biofluids (in particular blood) of individuals with subjective cognitive decline in incipient AD (A + SCD), including their longitudinal trajectories; in particular in their long-term longitudinal association with cognitive decline and brain alterations, and when specifically compared to both cognitively unimpaired individuals without cognitive decline (A + CU) and those with cognitive impairment in the presence of amyloid pathology (A + MCI) [8]. Investigations into molecular characteristics of SCD are essential to validate the concept of A + SCD as an indeed distinct disease stratum, serving as the transitional stage 2 between fully asymptomatic individuals (stage 1) and MCI (stage 3). This might allow to further strengthen the conceptual relevance of SCD in AD, and also to deliver specific molecular biomarker signatures - even in blood- for both prediction of clinical progression and stratification of participants for early intervention. This is of immediate importance because an increasing number of disease-modifying AD therapies is under approval or in development [4, 15, 16].

Blood-based biomarkers are ideally suited for tracking molecular changes longitudinally and predicting clinical outcomes in pre-dementia AD stages including SCD, given their extensive applicability, minimal invasiveness, and the ease with which they can be repeatedly assessed over time [17], in particular when compared to PET or CSF. Blood biomarker studies have demonstrated that - amongst other plasma tau markers - tau phosphorylated at threonine-181 (p181) specifically captures AD brain pathology, and can differentiate AD from other neurodegenerative diseases [2, 18]. Furthermore, plasma p181 has been shown to predict cognitive decline and progression from MCI to dementia [19]. Neurofilament light chain (NfL), a non-disease specific marker for the intensity of ongoing axonal damage, has also demonstrated potential as a blood-based biomarker in neurodegenerative diseases including AD [20, 21]. Elevated plasma NfL levels have been associated with cognitive decline and an increased risk for progression from MCI to dementia [22, 23].

In this study, we first classified individuals as amyloid-positive using the CSF Aβ 40/42 ratio. This was fundamental for accurately modeling the clinical AD continuum including SCD proposed by the NIA-AA framework, which requires the presence of amyloid pathology as a diagnostic biomarker [6]. Plasma p181 tau and NfL levels were then studied as stratification biomarkers for SCD along the AD clinical continuum, as well as biomarkers for conversion and progression. Specifically, we investigated whether these biomarkers for T (tau pathology) and N (neurodegeneration) within the ATN framework of AD pathology [6] and their changes over time could serve as molecular evidence for SCD as a distinct stage in the progression of AD. Additionally, we examined whether these biomarkers could help identify individuals at higher risk for future clinical deterioration. To this end, we assessed plasma p181 tau and NfL levels and their longitudinal trajectories in their association with hippocampal atrophy, prospective cognitive decline, and AD stage transition to MCI and dementia in a large prospective multi-center cohort of individuals with SCD. The observed changes were specifically contrasted to the alterations in both CU and MCI individuals, thus allowing to validate SCD as a distinct clinical and molecular stage in the pre-dementia AD continuum.

## Methods

### Participants

We analyzed data from 457 participants of the Longitudinal Cognitive Impairment and Dementia Study (DELCODE) of the German Center for Neurodegenerative Diseases (DZNE), of whom CSF data was available. DELCODE is an observational longitudinal memory clinic-based multicenter study carried out by DZNE associated university memory clinics in Germany. The ethical committees of all participating centers approved the protocol. The study was performed in accordance with the ethical standards as laid down in the 1964 Declaration of Helsinki and its later amendments or comparable ethics standards. All participants provided informed consent prior to study participation. A complete description of all inclusion and exclusion criteria has been published elsewhere [24]. In brief, all participants were 60 years or older and enrolled between 2014 and 2018. For this study, we included all patients from which plasma samples as well as baseline CSF were available. All participants classified as SCD (*n* = 210) presented to memory clinics through referral or self-referral with complaints of cognitive decline, and fulfilled the SCD research criteria (i) self-experienced decline in cognitive functioning, compared with a previously normal cognitive status, which is unrelated to an acute event; and (ii) normal performance on standardized tests used to classify MCI, corrected for age, sex, and education. Unimpaired cognition in objective testing was defined as a test performance better than −1.5 SD on all subtests of the Consortium to Establish a Registry for Alzheimer’s Disease (CERAD) neuropsychological test battery. The non-SCD cognitively unimpaired group (CU, *n* = 89) was recruited by advertisement, explicitly addressing individuals who felt healthy and without any relevant cognitive problems. Unimpaired cognition of CU individuals was verified according to the same criteria as above described for the SCD group. In addition, participants with amnestic MCI (*n* = 110) and mild dementia of Alzheimer type (DAT) (*n* = 48, MMSE ≥ 18 points) were recruited according to current research criteria for MCI and DAT (NIA-AA) [25, 26].

### Cognitive testing

At baseline and annual follow-ups a comprehensive cognitive test battery was applied by trained neuropsychologists at all sites [24, 27]. Alternate test versions of the FCSRT-IR and the ADAS Cog word lists and of the SDMT were applied, in order to reduce test-repetition effects. We calculated the Preclinical Alzheimer Cognitive Composite (PACC5), which has been extensively validated to detect subtle cognitive changes in individuals who are in the pre-dementia stage of Alzheimer’s disease [28, 29]. PACC5 was calculated as the average z-standardized performance in memory (FCSRT Free Recall and Total Recall), verbal episodic memory (Wechsler Memory Scale – Forth Edition (WMS-IV) Logical Memory Story B delayed recall), global cognition (MMSE), attention and processing (Symbol-Digit-Modalities Test), and verbal fluency (the sum of two category fluency tasks). Baseline mean and SD values of the CU group were used to derive the subtest z-scores. In CU and SCD, incident MCI was diagnosed by clinical consensus in individuals with evidence of longitudinal cognitive decline [30]. In MCI patients, incident dementia was diagnosed by the study physician according to established criteria.

### Magnetic resonance imaging

Magnetic resonance imaging (MRI), including up to 4 years longitudinal imaging, was carried out at nine imaging sites on Siemens 3T-MR-Scanners according the DZNE imaging protocol, and quality control process, as described previously [24]. Automatic hippocampal subfield segmentation was performed on high-resolution T2-weighted images, from which whole hippocampal volumes were derived using the Freesurfer image analysis suite [14], which is freely available for download online (http://surfer.nmr.mgh.harvard.edu/).

### Fluid biomarkers

#### CSF biomarkers

CSF Aβ42, Aβ40, and total-tau were analyzed centrally in one lab using commercial V-Plex ELISAs (Mesoscale Diagnostics, Rockville, USA); and CSF p181 with the Innotest Phospho-Tau(181 P) ELISA (Fujirebio Germany GmbH, Hannover, Germany). Independent reference samples were used to control assay performance. Cut-off values for both CSF Aβ42/Aβ40 ratio and tau were determined from the DELCODE data set using Gaussian mixture modelling using the R package flexmix, version 2.3–15 [14]. The following cut-offs were applied to indicate Alzheimer’s pathological changes: CSF Aβ42/40 ratio ≤ 0.08 and total-tau ≥ 510.9 pg/mL. The cut-off of the Aβ42/40 ratio was used to define amyloid positivity. We aimed to examine the trajectory of plasma biomarkers and their association with clinical measures in individuals across the AD pre-dementia continuum. Following the 2018 NIA-AA research framework, Aβ biomarker positivity indicates whether an individual falls in the AD continuum. Participants were thus grouped based on their CSF amyloid levels, including: participants with amyloid deposition, but without cognitive impairment and without subjective cognitive decline (A + CU); participants with amyloid deposition and subjective cognitive decline (A + SCD); and participants with amyloid deposition and mild cognitive impairment (A + MCI). Clinical groups without evidence of Alzheimer’s pathology (A-CU, A-SCD, and A-MCI) were included for comparison (Table 1 and Figure S1A+B). DAT patients had to fulfill CSF criteria for amyloid and tau positivity as defined above.

**Table 1:** Demographics and clinical characteristics.

CSF β-amyloid positivity	CU		SCD		MCI		AD	*p* value
	A + CU	A-CU	A + SCD	A-SCD	A + MCI	A-MCI		
NIA-AA stage	I		II		III		IV+	
N	24	65	81	129	69	41	48	
Mean follow-up time, mo	43.8 ± 4.1	47.5 ± 2.1	36.0 ± 2.0	36.2 ± 1.6	33.4 ± 2.7	29.6 ± 3.2	24.4 ± 2.7	0.070
Age, y (at baseline)	70.1 ± 1.1	68.6 ± 0.6	73.3 ± 0.6	70.3 ± 0.5	73.6 ± 0.6	70.5 ± 0.8	75.3 ± 0.9	0.017
Sex F/M, n	8/16	27/38	28/53	62/67	35/34	11/30	32/16	0.097
Education, y	14.8 ± 0.6	14.4 ± 0.3	14.9 ± 0.3	14.9 ± 0.3	13.5 ± 0.4	14.6 ± 0.4	12.8 ± 0.4	0.009
CSF Aβ40 in pg ml^−1^	8402 ± 422	8806 ± 309	8300 ± 229	8462 ± 199	8332 ± 270	7712 ± 390	8851 ± 301	0.901
CSF Aβ42 in pg ml^−1^	542 ± 29	936 ± 34	498 ± 18	946 ± 34	432 ± 17	848 ± 52	404 ± 18	0.003
CSF Aβ 42/40 ratio	0.066 ± 0.003	0.107 ± 0.002	0.060 ± 0.001	0.111 ± 0.001	0.052 ± 0.002	0.108 ± 0.002	0.046 ± 0.001	<0.001
CSF Tau in pg ml^−1^	433 ± 41	343 ± 17	490 ± 23	290 ± 10	634 ± 38	376 ± 28	961 ± 44	<0.001
PACC5, z-score	−0.045 ± 0.109	0.182 ± 0.056	−0.318 ± 0.069	−0.071 ± 0.056	−1.857 ± 0.121	−1.217 ± 0.137	−3.673 ± 0.246	<0.001
MMSE total score	29.3 ± 0.1	29.4 ± 0.1	29.1 ± 0.1	29.1 ± 0.1	27.2 ± 0.2	28.1 ± 0.3	23.0 ± 0.4	<0.001

Data are mean ± SEM unless otherwise specified. *p*-values are derived from Chi-square (sex) or Kruskal-Wallis H tests comparing A + CU, A + SCD, and A + MCI.

*AD* Alzheimer’s disease dementia, *CSF* cerebrospinal fluid, *CU* cognitively unimpaired, *F* female, *M* male, *MCI* mild cognitive impairment, *MMSE* mini-mental state examination, *NIA-AA* framework suggested by the national institute on aging and the Alzheimer’s association for the diagnosis and staging of Alzheimer’s disease, *PACC5* preclinical Alzheimer’s disease cognitive composite, *SCD* subjective cognitive decline.

#### Plasma biomarkers

Plasma levels of p181 and NfL were quantified using Simoa assays. All assays were performed by the same operator, and conducted on Quanterix HD-1 and HD-X instruments (Quanterix, Billerica, MA). Simoa NF-light Advantage (Quanterix, Billerica, MA, USA) and pTau-181 advantage version 2 kits were used according to the manufacturer’s instructions. Samples were diluted 1:4 in sample buffer and analyzed in technical duplicates. The lower limit of quantitation (LLoQ) was defined as the lowest standard: (i) with a signal higher than the average signal for the blank plus 9 SDs, and (ii) allowing a percent recovery ≥ 100 ± 20%. For p181 and NfL, the LLoQ between runs was 0.77 and 1.80 pg/mL, respectively. Two internal control samples were assessed both at the start and end of each assay run to determine repeatability and inter-assay variability. The % repeatability for p181 and NfL was 5.6% (sample 1) and 7.6% (sample 2), and 9.2% (sample 1) and 4.6% (sample 2), respectively. The inter-assay variance was 12.4% (sample 1) and 11.8% (sample 2), and 9.1% (sample 1) and 6.5% (sample 2), respectively. Samples were excluded from further analysis (2.4% for p181, and 3.8% for NfL), if the %CV was > 20% between two technical replicates or if measurements were available of only one technical replicate.

### Statistical analysis

**Fig. 1:**
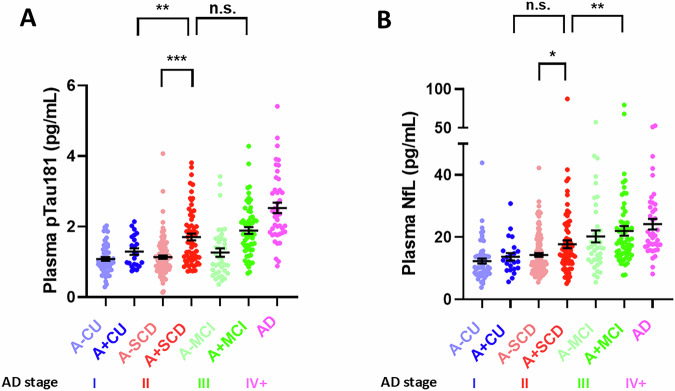
Plasma p181 and NfL are elevated in A+SCD. CU (blue), SCD (red), and MCI (green) individuals were stratified by CSF-amyloid positivity (A+ vs A-), and their baseline plasma analyzed using Simoa assays to detect **A** p181 and **B** NfL. Plasma from AD patients (purple) was included as a reference for both biomarkers. The respective stage in the AD continuum, operationalized using clinical assessment combined with CSF amyloid-positivity, is indicated below the graph (I: A + CU, II: A + SCD, III: A + MCI, IV + : AD). In both graphs, each point represents an individual subject, and means ± SEM are indicated. Differences between groups were assessed using Welch-ANOVA followed by Dunnett’s post hoc test (for p181 data) or Kruskal Wallis H test followed by Dunn’s post hoc test (for NfL data) for the contrasts indicated. *p < 0.05, **p < 0.01, ***p < 0.001, n.s., non-significant. **A** Plasma p181 levels are elevated in A + SCD compared to A + CU and A-SCD, while levels do not differ between A + SCD and A + MCI. **B** Plasma NfL levels show a trend towards increased levels in A + SCD compared to A + CU, and are significantly increased in A + SCD compared to A-SCD. Plasma NfL levels are then further elevated in A + MCI vs A + SCD.

Statistical analyses were carried out using GraphPad Prism, version 10.1.2 (LaJolla, CA, USA) and Stata, version 17.0 (College Station, TX, USA). Normal distribution was assessed by visual inspection of histograms and Quantil-Quantil-plots. Differences of biomarker levels between groups were assessed using Welch-ANOVA followed by Dunnett’s post hoc test (for normally distributed p181 data in Fig. 1 and CSF data in Supplementary Fig. 1) or Kruskal Wallis H test followed by Dunn’s post hoc test (for non-normally distributed NfL data in Fig. 1) for the indicated contrasts, which were determined a priori based on the study’s research question. For Table 1, *p*-values were derived from Chi-square (sex) or Kruskal-Wallis H tests comparing A + CU, A + SCD, and A + MCI. Linear mixed effect (LME) models were fitted to analyze plasma p181 and NfL trajectories over time in CU, SCD, and MCI individuals stratified by amyloid positivity. LME models were adjusted for age and gender, and included an interaction between time and diagnostic group, as well as random intercepts and slopes nested within subject. To study associations of amyloid-positivity or baseline plasma levels (categorized by 3-quantiles) with longitudinal PACC5 and hippocampal volume (average of left and right sight), LME models were fitted including an interaction factor between the categorical variable of interest and time. Models were adjusted for age and sex, and included random intercepts and slopes nested within subject. For cognition, we also included years of education as covariate. Total brain volume was incorporated as a covariate for hippocampal volume analysis. Linear additivity was assessed by visual inspection of the residuals vs. fitted plot. *p*-values were adjusted for multiple comparisons through false discovery rate (FDR) using the Benjamini-Hochberg correction [31, 32] (if more than two comparisons (plus a global joint hypothesis test) were examined for an interferential hypothesis), and were considered significant at *p* ≤ 0.05, two-tailed. To assess associations between amyloid positivity and plasma levels of p181 and NfL (categorized by 2-quantiles) and risk of incident MCI or dementia, we used Kaplan-Meier survival analysis and cox proportional hazards regression. The proportionality of hazards was assessed using the Schoenfeld residuals.

## Results

### A+SCD individuals exhibit a distinct plasma p181 trajectory and increased plasma NfL levels

Cross-sectional plasma p181 levels were elevated in A + SCD compared to A + CU (1.7 ± 0.1 vs 1.3 ± 0.1 pg/mL; *p* = 0.009) and to A-SCD (1.1 ± 0.0 pg/mL; *p* < 0.001) (Fig. 1A), while there was no difference of A + SCD compared to A + MCI (1.9 ± 0.1 pg/mL; *p* = 0.453) (for comparison to CSF p181 levels, see Supplement 1).Plasma NfL levels were increased in A + SCD compared to A-SCD (17.7 ± 1.3 vs 14.3 ± 0.6 pg/mL; *p* = 0.022). Compared to A + CU, plasma NfL levels in A + SCD showed a numeric (Fig. 1B) but statistically non-significant elevation (17.7 ± 1.3 vs 13.7 ± 1.2 pg/mL; *p* = 0.276), and NfL levels were then further elevated in A + MCI (22.0 ± 1.6 pg/mL) compared to A + SCD (*p* = 0.008) (for stepwise increase pattern across the three pre-dementia AD stages, see Fig. 1B).

**Fig. 2:**
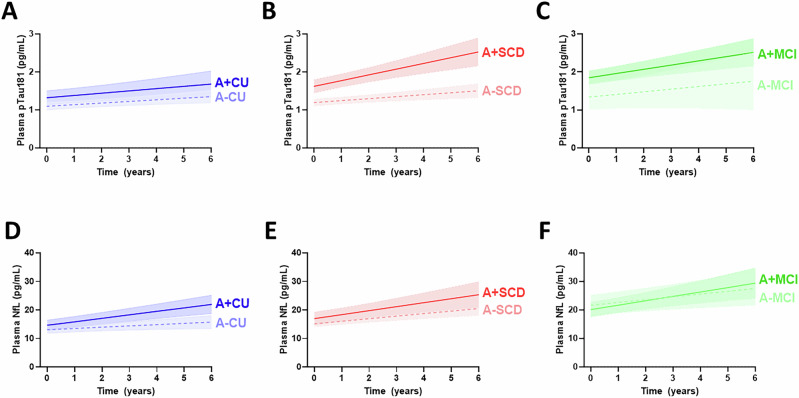
Plasma p181 levels increase at a higher rate over time in A+SCD and A+MCI compared to A+CU, while plasma NfL levels rise at a similar rate in A+CU, A+SCD, and A+MCI. Plasma p181 and NfL were measured in plasma samples collected at ~1 year intervals from **A**+**D** A + CU and A-CU, **B**+**E** A + SCD and A-SCD, and **C**+**F** A + MCI and A-MCI. **A**+**B** Plasma p181 levels in A + SCD, compared to each A + CU and A-SCD increase more steeply over time. **C** The rate of change is then kept in MCI. **D–F** Changes of plasma NfL levels over time are similar in A + CU, A + SCD, and A + MCI, which leads to elevated NfL in A + SCD and A + MCI compared to A + CU individuals. Estimated trajectories for CU (blue), SCD (red), and MCI (green) are drawn using mixed-effects modelling with an interaction term for time and amyloid-positivity, and adjusted for age at baseline, and sex. Shaded areas indicate 95% confidence intervals.

Analysis of longitudinal change demonstrates that plasma p181 levels increase at a faster rate over time in A + SCD (0.15 pg/mL/year) compared to both A + CU (0.06 pg/mL/year) (slope A + SCD vs A + CU, *p* = 0.044) and A-SCD (0.05 pg/mL/year) (slope A + SCD vs A-SCD, *p* = 0.016) individuals (Fig. 2), indicating that A + SCD individuals have a distinct trajectory of molecular ptau pathology, which is different from both A + CU and A-SCD. This accelerated rate of change, starting off in the SCD stage, is then sustained in A + MCI (slope A + SCD vs A + MCI, *p* = 0.308).

Longitudinal changes in plasma NfL levels were similar in A + CU (1.22 pg/mL/year), A + SCD (1.40 pg/mL/year), and A + MCI (1.54 pg/mL/year) individuals (slope A + SCD vs A + CU, *p* = 0.790; slope A + SCD vs A + MCI, *p* = 0.790) in a linear mixed model adjusted for age and gender, given the known association of age and NfL levels [33], indicating a similar axonal turnover rate across the pre-dementia AD continuum (slope A + CU vs A + SCD vs A + MCI, *p* = 0.765).

### Baseline plasma p181 levels predict future cognitive decline in A+SCD

**Fig. 3:**
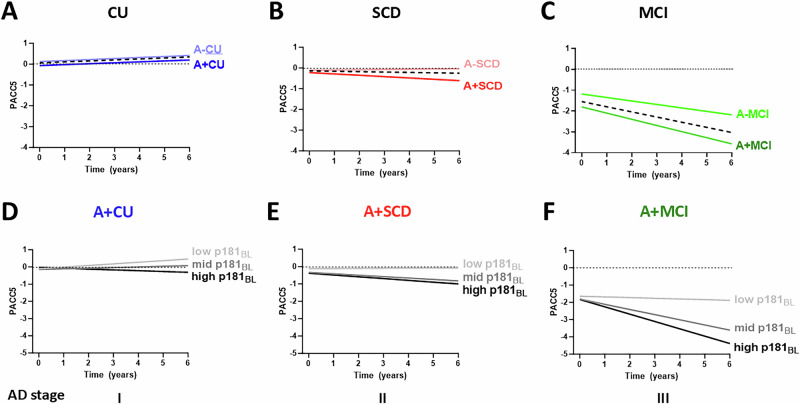
Longitudinal cognitive decline in A+SCD is predicted by higher plasma p181 levels at baseline. Longitudinal trajectories of the PACC5 score of **A** CU (blue), **B** SCD (red), and **C** MCI (green) individuals, stratified by CSF amyloid-positivity are displayed. Dashed black lines indicate the trajectories for the clinical groups (CU, SCD, MCI) irrespective of the CSF amyloid status. **A**–**C** The rate of cognitive deterioration over time, as measured by PACC5, is increased in SCD compared to CU, largely driven by the worse performance of A + SCD individuals. The observed cognitive trajectory in A + SCD is then accelerated further in A + MCI. In panel **D**–**F**, baseline (BL) plasma p181 levels of the A + CU, A + SCD, and A + MCI group, categorized into low (light grey), mid (medium grey), and high (dark grey) levels using 3-quantiles, are used to predict longitudinal performance on PACC5. **D**–**F** Future cognitive decline observed in the A + SCD group is associated with high p181 levels at baseline. This predictive association is not seen to the same extent in the A + CU group. Once this association is started in the A + SCD, it is then maintained in the A + MCI group. Trajectories were derived from linear mixed models with an interaction term for time and baseline p181 levels, and adjusted for age at baseline, sex, and years of education.

The rate of cognitive decline over time, as assessed through longitudinally measured PACC5, was increased already in individuals with SCD (slope SCD vs CU, *p* = 0.001), with this cognitive trajectory mainly driven by the cognitive decline in A + SCD (−0.06 ± 0.03 units/year), whereas A-SCD individuals showed no cognitive deterioration (+0.01 ± 0.02 units/year) (slope A + SCD vs A-SCD, *p* = 0.022, Fig. 3). In contrast to SCD, no cognitive deterioration could be observed in the CU group (+0.05 ± 0.01 units/year), also not in the A + CU group (+0.04 ± 0.02 units/year), as shown before in this cohort [14]. The slight PACC5 performance increase over time in CU and in A-SCD likely results from residual test-repetition effects, as not all PACC5 subtests had alternate versions. However, decline started off in the A + SCD stage, and was accelerated further in A + MCI (−0.30 units/year, slope A + MCI vs A + SCD, *p* = 0.005).

In the A + SCD group, higher plasma p181 levels at baseline were associated with cognitive decline (*p* = 0.036). In contrast, this association was not observed to a similar extent in the A + CU group. A standardized annual PACC5 change of −0.10 ± 0.03 was noted in individuals with A + SCD exhibiting the highest p181 levels, in contrast to an annual change of only −0.05 ± 0.05 for the A + CU group with the highest p181 levels. Once starting off in the A + SCD group, the association between higher baseline p181 levels and subsequent cognitive deterioration is then maintained and further accelerated in the A + MCI group (Fig. 3). In contrast to p181 levels, baseline NfL levels were associated with cognitive decline in A + MCI individuals, but not yet in A + SCD individuals (Figure S2).

We also examined hippocampal volume loss in the AD pre-dementia stages, including up to 4 years longitudinal volumetric MRI, in relation to blood biomarker levels. At baseline, hippocampal volumes were lower in SCD compared to CU, and further reduced in MCI (Figure S3). In A + MCI, but not yet in A + SCD, higher baseline ptau181 levels (Figure S3), were numerically associated with lower hippocampal volume, while NfL levels (Figure S4) did not show any trend of association. Further discussion on these findings is provided in the Supplement.

### Baseline levels of plasma p181 predict progression to MCI in A+SCD

**Fig. 4:**
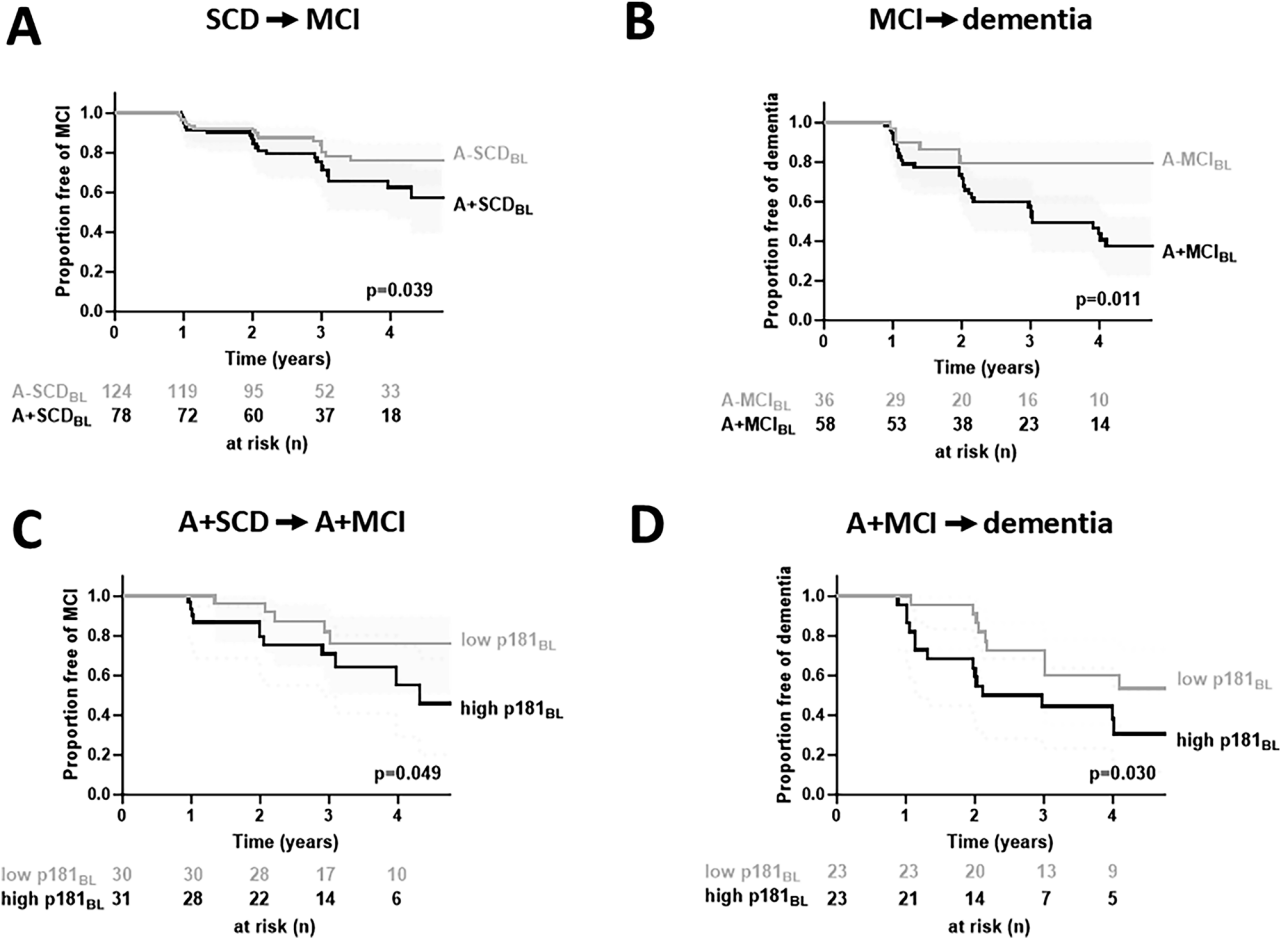
A+SCD compared to A-SCD individuals have a higher risk for conversion to the MCI stage, which is further increased in the presence of higher baseline levels of p181. Survival curves for progression from **A** SCD to MCI, and **B** MCI to dementia, stratified by CSF amyloid-positivity are indicated. **A** SCD and **B** MCI individuals have a higher risk of progression to MCI and AD, respectively, in the presence of amyloid deposition. In panel **C,** **D**, progression from **C** A + SCD to MCI, **D** and A + MCI to dementia among individuals with high (first and second quartile, in black) versus low (third and fourth quartile, in light grey) baseline plasma levels of p181 are shown. The x-axis shows the time of follow-up from diagnosis of SCD or MCI, while the y-axis is the fraction of patients free of MCI or dementia at given time-points. Values below each graph indicate the number of subjects free of MCI/dementia at 0, 1-, 2-, 3-, and 4-years follow-up from first detection of SCD/MCI. Higher baseline plasma p181 levels predict the conversion of **C** A + SCD to MCI, and **D** A + MCI to dementia.

A total of 202 SCD and 94 MCI individuals were evaluated for clinical progression to MCI and dementia at follow-up, respectively. Of those, 44 SCD (21.8%) and 37 MCI (39.4%) converted to MCI or dementia, respectively. A + SCD individuals had a higher risk of progression to MCI than A-SCD individuals (hazard ratio 1.9 ± 0.6, *p* = 0.039) (Fig. 4). In a mean follow-up time of 3.0 ± 0.1 years, 29.5% of A + SCD converted MCI, compared to 16.9% in the A-SCD group. Similarly, A + MCI individuals had a higher risk of progression to AD than A-MCI individuals (hazard ratio 3.1 ± 1.4, *p* = 0.011) (Fig. 4).

Higher baseline p181 levels were significantly associated with future conversion from SCD to MCI (hazard ratio = 2.9 ± 1.5, *p* = 0.049) in A + SCD. Similarly, higher baseline p181 levels were also significantly associated with future progression from A + MCI to dementia (hazard ratio = 2.5 ± 1.1, *p* = 0.030). Baseline NfL levels did not significantly predict clinical AD stage transition (Figure S5).

## Discussion

This prognostic study investigated the potential of blood-based biomarkers, specifically plasma p181 and NfL, to reveal molecular changes in individuals with SCD. It would provide molecular blood biomarker support for SCD as a transitional stage 2 between the fully asymptomatic stage 1 and stage 3 according to the NIA-AA Research Framework; plus – at the same time- pave the path for a blood-based signature of the ATN AD scheme (with p181 representing T/tau, and NfL representing N/neurodegeneration), thereby also facilitating the NIA-AA’s 2023 proposal to include blood-based biomarker in this classification scheme [34]. Moreover, we also examined their capacity to predict both future cognitive decline and AD stage transition to MCI.

### Plasma p181 is elevated in A+SCD

Our results reveal an elevation in baseline plasma p181 levels in A + SCD in comparison to A + CU, and this elevation also increases at a higher rate over time compared to A + CU individuals. While individuals in both A + CU and A + SCD groups lack cognitive impairment and, consequently, appear indistinguishable in neuropsychological assessments, the biomarker trajectories of plasma p181 in A + SCD more closely resemble those observed in A + MCI than in A + CU. This suggests a pertinent molecular progression along the pre-dementia AD continuum in individuals with SCD (stage 2), to a similar extent as in A + MCI (stage 3), but distinct from those without subjective cognitive decline (A + CU, stage 1). Prior research has shown a rise in plasma phospho-tau including p181 along the clinical AD continuum [35], with elevated levels observed in AD dementia compared to both MCI and CU [19, 36]. It has been proposed that the release of extracellular soluble tau is related to early dysregulation in neuronal tau metabolism due to early Abeta pathology, and (in later stages) tau fibril formation [35]. However, when assessing plasma ptau levels in AD and MCI patients, the comparisons were largely made with cognitively unimpaired individuals or healthy controls. Furthermore, SCD patients were often times placed within either the CU or MCI groups, precluding to determine the role of biomarkers in this particular pre-dementia AD stratum [19, 20]. Notably, to our knowledge no study has so far explored longitudinal ptau biomarker trajectories in A + CU in direct comparison to A + SCD groups. This study addressed this gap and enabled us to demonstrate a unique molecular ptau trajectory in individuals with A + SCD. The observed elevation in p181 levels in A + SCD, before the onset of objective cognitive impairment, underscores the potential utility of this blood-based biomarker in identifying individuals at this very early stage of the pre-dementia AD continuum.

### Plasma NfL adds further support for SCD stage an intermediate AD stage between CU and MCI, with incipient axonal degeneration

Plasma NfL levels tended to be numerically increased levels in A + SCD compared to A + CU, and were - in particular- significantly increased in A + SCD compared to A-SCD. Once starting off at the A + SCD stage, plasma NfL levels then increased further in the A + MCI stage. Within the SCD cohort, these findings suggest that axonal degeneration is already detectable and peripherally captured in A + SCD individuals. This molecular biomarker signature of early incipient neurodegeneration from the SCD stage onwards in the clinical AD continuum further supports A + SCD as an intermediate between fully asymptomatic A + CU (=AD stage 1) and A + MCI (=AD stage 3). In contrast, NfL biomarker analysis does not support A-SCD as a neurodegenerative state. This differs from the MCI stage, where individuals –irrespective of their amyloid status – show a molecular biomarker signature of axonal degeneration (i.e. increased NfL levels in both A+ and A-MCI, without statistical difference). The difference between A-MCI and A + MCI was not significant for NfL, although A + MCI participants are older. Diagnostic follow-up of the A-MCI group revealed that 10 out of 38 patients received a clinical diagnosis of dementia or Parkinson disease later on; these neurodegenerative diseases have probably – as likely often the case in A-MCI – raised the NfL levels in the A-MCI group at baseline, making them indistinguishable from the levels in the A + MCI group. This sequential elevation in NfL levels across the pre-dementia AD stages further supports the idea of a continuous biological process underlying the progression of AD along the proposed clinical continuum. This is further supported by the fact that longitudinal changes in plasma NfL levels were similar in A + CU (stage 1), A + SCD (stage 2) and A + MCI (stage 3), indicating a similar axonal turnover rate across the pre-dementia AD continuum.

In contrast to the distinctive plasma p181 trajectory observed in individuals with A + SCD, alterations in NfL levels at baseline and over time between A + CU and A + SCD are, however, more nuanced, merely reflecting a trend - and likely a lesser degree of neurodegeneration at these very early stages of pre-dementia AD compared to MCI.

### Plasma p181 predicts future cognitive decline in A+SCD

The rate of cognitive decline over time, as assessed by PACC5, was accelerated in individuals with SCD, primarily driven by the poorer performance in A + SCD. In contrast to SCD, the CU group exhibited no cognitive decline, regardless of amyloid positivity. This cognitive trajectory, starting off in A + SCD individuals, was then further accelerated in A + MCI. These findings, supporting earlier reports from the DELCODE cohort [14], indicate that A + SCD shows a distinct cognitive trajectory different from CU, including also in particular from A + CU. This degree of future cognitive decline in the A + SCD group could be predicted by plasma p181 levels at baseline. In contrast, this predictive association was not seen to the same extent in the A + CU group. Once this association is started in the A + SCD, it is then maintained in the A + MCI group. Taken together, this thus adds further support for A + SCD as an intermediate stage between A + CU and A + MCI.

This is the first study showing a specific association of plasma p181 with longitudinal cognition in A + SCD vs. A + CU individuals. In addition, our data also extend and specify previous studies that had indicated a relationship between baseline phosphorylated tau measures in CSF and future cognitive decline in SCD [13, 37]. The now identified predictive association of elevated plasma p181 levels in A + SCD for future decline in PACC5 holds important clinical significance. These findings indicate that plasma p181 levels may function as a predictive marker for a critical clinical functionality - namely future cognitive decline specifically within the A + SCD group. These group level results indicate that plasma p-tau181 might help identify individuals who may be at an increased risk of future cognitive decline, even at this early stage of the pre-dementia AD continuum; however, further validation is needed to establish its predictive accuracy and clinical utility on the individual subject level.

### Plasma p181 predicts future clinical stage transition in A+SCD

The overall SCD group showed a conversion rate to MCI of 21,8% (17,0% for A-SCD and 29,5% for A + SCD) in a mean follow-up time of 3 years. The finding of 20% SCD-to-MCI converters corroborates and extends previous longitudinal studies [8, 11], suggesting that SCD can herald progressive worsening of cognitive functions for a considerable share, but not *all* SCD subjects. Numerous health issues beyond neurodegenerative diseases - including mental health conditions such as depression, anxiety, temporal stress, or even fatigue - have the potential to cause SCD. This underscores the necessity of substantiating SCD through, as shown here, - ideally peripheral (i.e. blood-based) capturable- molecular biomarkers that capture potential underlying amyloid pathology and/or neurodegeneration.

The risk of conversion to MCI was particularly increased in the A + SCD group (29,5% converters). This finding substantiates the clinical relevance of A + SCD as risk stage for further disease progression along the pre-dementia AD continuum. The average time to MCI in converters was only 2.3 years, underscoring the clinical relevance of A + SCD for designing trials for early intervention.

This higher risk of conversion to MCI was associated with higher baseline levels of plasma p181 in A + SCD, i.e. plasma p181 levels allow to predict future clinical decline and conversion to the MCI stage. This predictive association was also observed for progression to dementia in the A + MCI group. This indicates the use of plasma p181 in A + SCD as a stratification biomarker for identifying which SCD subjects might be at higher risk of converting to MCI. In addition, it suggests that plasma p181 tau – with treatment-responsivity now increasingly demonstrated [4, 38]- might also serve a as a potential therapy response biomarker for future treatment trials already targeting the SCD stage charged with clinical meaningfulness – as highly requested for biomarkers by the FDA [5] - as it reflects conversion to a substantially different stage in the clinical dementia continuum.

### Plasma p181 for identification of fast decliners in A+SCD to facilitate trials with disease-modifying treatments

The cognitive decline in A + SCD is substantially less severe than in A + MCI [13, 39]. This presents a challenge for conducting trials with disease-modifying treatments in SCD, as there is often no relevant cognitive decline in the placebo-treated group. This highlights the urgent need to identify biomarkers that allow stratification of decliners within the A + SCD group, where such trials would be feasible. Here, we propose that plasma ptau181 could assist in identifying a subset within the A + SCD group who may experience faster cognitive decline, which may help to enrich and refine participant selection for disease-modifying treatment trials within the pre-dementia Alzheimer’s disease window.

### Limitations

Our study has several limitations. First, our biomarker results on the prediction of cognitive decline are based on interferences on a group-level, which restricts their use in memory clinics to predict cognitive trajectories at an individual level. Upon prospectively acquiring more data from individuals who have progressed from a cognitively unimpaired state through the successive stages of the clinical Alzheimer’s disease continuum in the ongoing DELCODE study, future analyses are warranted to allow focussing on individual longitudinal biomarker trajectories and assessing the relevance of both intra- and inter-individual variances in robustly forecasting cognitive traits. Secondly, the representativeness for the general population is limited because the individuals were recruited from specialized memory clinics where they sought help because of memory complaints. However, this characteristic is linked to a higher likelihood of including participants who will undergo objective cognitive decline in SCD, compared to those with SCD who do not seek medical assistance [8, 40]. Moreover, we consider this group to be particularly motivated to participate in research and intervention, thus representing an ideal target population for clinical trials and early treatment. The lack of significance of MRI findings in our study may stem from the constrained observational follow-up period - while longitudinal capture of 4 years follow-up biomarker and MRI volumetric data already covers a relevant timeframe, it might be too short to identify robust associations. While acknowledging this constraint, we recognize the potential for further investigation into the predictive abilities of brain atrophy in relation to blood-captured molecular pathology, particularly as extended observational follow-up times become accessible. Additionally, measurements of other plasma phospho tau species, notably p217, were not available during the analysis of this study. However, they will be assessed in future studies within this cohort.

## Conclusion

In summary, our research offers strong support for the significance of plasma p181 levels as a molecular marker of AD disease progression within A + SCD, identifying it as a distinct pre-dementia stage of Alzheimer’s disease (stage 2). This stage falls between the asymptomatic stage 1 (A + CU) and the prodromal stage 3 (A + MCI) within the NIA-AA clinical AD continuum framework. The predictive capability of plasma p181 for future cognitive deterioration and the transition to MCI at such an early phase of the disease, where not only cognitive functions are still largely intact, but also axonal degeneration is yet still just incipient (as indicated by our NfL findings) may offer significant potential for the early detection and timely intervention.

## Supplementary information

Supplemental Material

## Data Availability

De-identified data are available from the corresponding author on reasonable request. Contact the corresponding author for more information.
