# Supplementary material for: Blood biomarkers confirm subjective cognitive decline (SCD) as a distinct molecular and clinical stage within the NIA-AA framework of Alzheimer´s disease
Source: Mol Psychiatry. 2025 Apr 18;30(7):3150–9. doi: 10.1038/s41380-025-03021-0 (PMC12185333; doi:10.1038/s41380-025-03021-0)
Supplement: Supplementary file 1 — Supplemental Material [file 41380_2025_3021_MOESM1_ESM.pdf]

Supplement

Supplemental results and discussion

CSF p181 mirrors plasma p181 in A+SCD vs A-SCD, but not in A+CU vs A+SCD

The cross-sectional biomarker findings for CSF p181 were similar to those for plasma p181 (Fig. S1C); however, CSF p181 could not distinguish between the A+CU and A+SCD groups. Conversely, it showed an increase in the A+MCI group compared to the A+SCD group, where plasma p181 levels had already flattened out. This suggests that plasma p181 may be more sensitive in detecting p-tau pathology in A+SCD. However, further studies are needed to replicate these results.

Baseline levels of plasma p181 are associated with hippocampal volume loss in A+MCI, but not in A+SCD

Hippocampal volumes at baseline were lower in SCD compared CU (3061±21 vs 3148±28 mm^3, p=0.016), and even further reduced in MCI individuals (2885±36 mm^3, p<0.001, Fig. S2). Within A+MCI, but not yet in the A+SCD, baseline ptau181 levels showed a qualitative association with lower hippocampal volume (A+MCI: p=0.119, A+SCD: p=0.439). Baseline NfL levels were not associated with lower hippocampal volume in any of the pre-dementia stages (Fig. S3).

Supplemental figure captions

Supplemental Figure 1. The CSF Aβ42/40 ratio separates A+ from A- clinical groups, and CSF p181 replicates findings in plasma p181 in A+SCD vs A-SCD, but not between A+CU vs A+SCD. Baseline CSF of CU (blue), SCD (red), MCI (green), and AD (purple) of the same patients as in Figure 1A was analyzed using ELISAs to measure **(A)** the Aβ42/40 ratio **(B)** mid-region tau

(=total tau) and **(C)** p181. In both graphs, each point represents an individual subject, and means ± SEM are indicated. Differences between groups were assessed using Welch-ANOVA followed by Dunnetts post hoc test for the contrasts indicated, *p<0.05, **p<0.01, ***p<.001, ****p<0.0001, ns, non-significant. **(A)** The CSF Aβ42/40 ratio, **(B)** CSF mid-region tau, and **(C)** CSF p181 separates A-SCD from A+SCD and is increased in A+MCI compared to A+SCD, but not in A+SCD vs A+CU.

Supplemental Figure 2. Plasma NfL levels are not associated with future cognitive decline in A+SCD. (C) Baseline plasma NfL levels predict future cognitive decline only in the MCI stage, lacking notable relevance in both A+CU **(A)** and A+SCD **(B)**. Trajectories are modeled as outlined in Figure 3, incorporating an interaction term of time and baseline NfL levels.

Supplemental Figure 3. Hippocampal volume loss in A+MCI is associated with higher baseline levels of plasma p181. Longitudinal trajectories of whole hippocampal volumes of **(A)** CU (blue), **(B)** SCD (red), and **(C)** MCI (green) individuals, stratified by CSF amyloid-positivity are depicted. Dashed black lines indicate the trajectories for the clinical groups irrespective of the CSF amyloid status. Reduced hippocampal volumes are noted in SCD compared to CU individuals. Subsequently, hippocampal volumes experience further reduction in the MCI stage, reaching their lowest levels in A+MCI. In panel **(D – F)**, baseline (BL) plasma p181 levels of the A+CU, A+SCD, and A+MCI group, categorized into a low (light grey), mid (medium grey), and high (dark grey) tertile, were used to predict whole hippocampal volumes at baseline and longitudinally over the study period of four years. In the A+MCI group, higher baseline ptau181 levels are qualitatively associated with reduced hippocampal volume. Trajectories are derived from linear mixed models with an interaction term for time and baseline p181 levels, and adjusted for age at baseline, sex, and total brain volume.

Supplemental Figure 4. Hippocampal volume loss is not correlated with baseline NfL. (A - C) Higher NfL levels at baseline are not associated with lower hippocampal volume in pre-dementia AD stages. Trajectories are modeled as outlined in Supplemental Figure 3, but with an interaction term of time and tertiles of baseline NfL levels.

Supplemental Figure 5. Higher NfL levels at baseline do not predict clinical stage transition in A+SCD and A+MCI. Survival curves for **(A)** A+SCD and **(B)** A+MCI indicate, that higher NfL levels to not predict future progression to **(A)** MCI or **(B)** dementia. Statistical interference and labeling of the graphs are as outlined in Figure 4.

SFigure 1

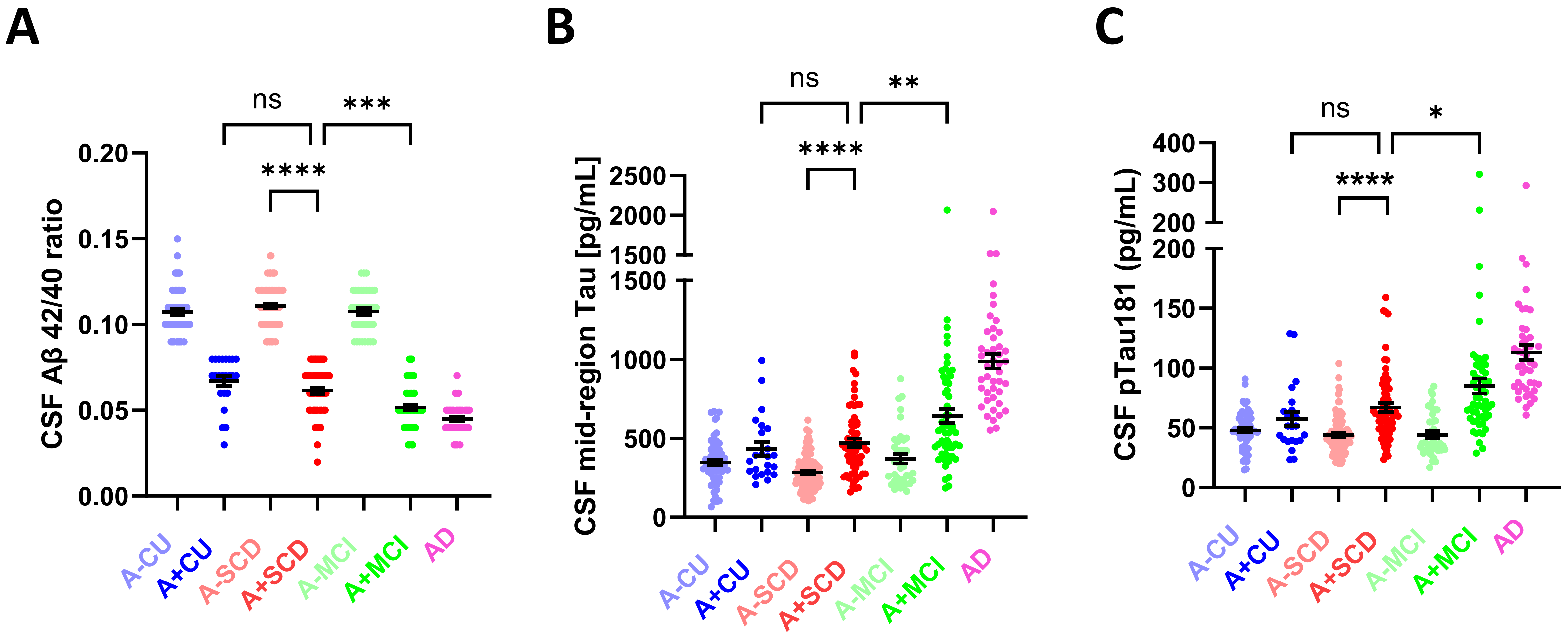
A
B
C
CSF Aβ 42/40 ratio
0.20
0.15
0.10
0.05
0.00
ns
***
****
CSF mid-region Tau [pg/mL]
2500
2000
1500
1500
1000
500
0
ns
**
****
CSF pTau181 (pg/mL)
400
300
200
200
150
100
50
0
ns
*
****
A-CU
A+CU
A-SCD
A+SCD
A-MCI
A+MCI
AD

SFigure 2

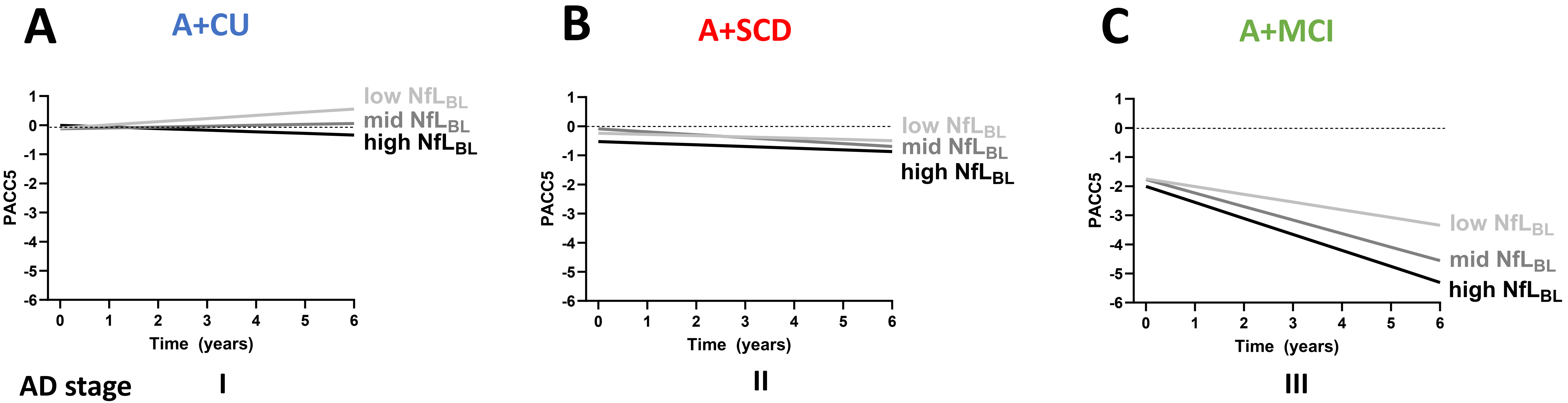
A
A+CU
B
A+SCD
C
A+MCI
PACC5
Time (years)
low NfL_BL
mid NfL_BL
high NfL_BL
AD stage
I
II
III

SFigure 3

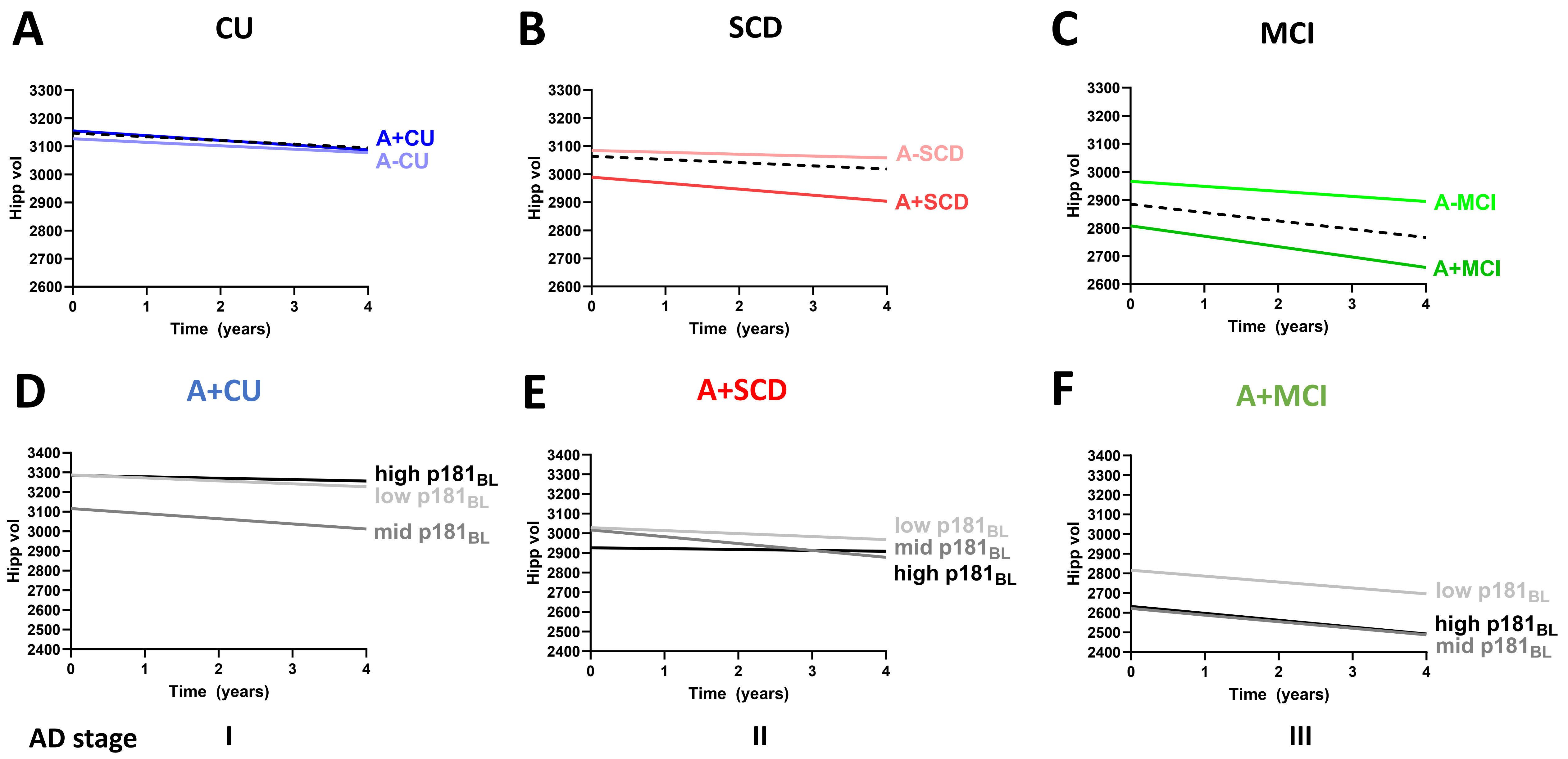
A
CU
3300
3200
3100
3000
2900
2800
2700
2600
Hipp vol
0
1
2
3
4
Time (years)
A+CU
A-CU
B
SCD
A-SCD
A+SCD
C
MCI
A-MCI
A+MCI
D
A+CU
3400
2500
2400
high p181BL
low p181BL
mid p181BL
E
A+SCD
F
A+MCI
AD stage
I
II
III

SFigure 4

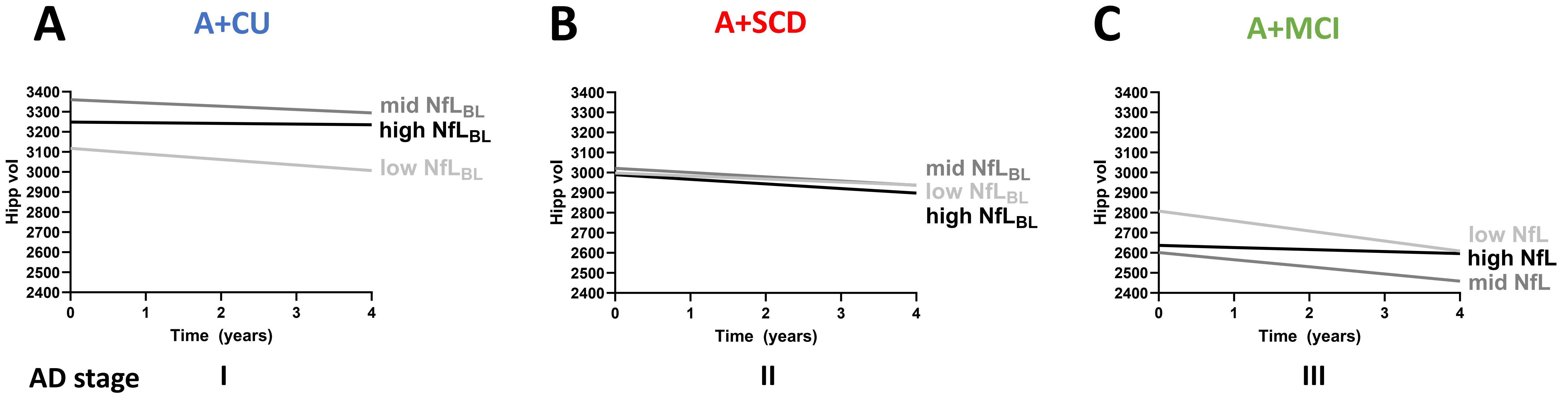

A
A+CU
3400
3300
3200
3100
3000
2900
2800
2700
2600
2500
2400
Hipp vol
0
1
2
3
4
Time (years)
mid NfL$_{BL}$
high NfL$_{BL}$
low NfL$_{BL}$
B
A+SCD
Hipp vol
Time (years)
mid NfL$_{BL}$
low NfL$_{BL}$
high NfL$_{BL}$
C
A+MCI
Hipp vol
Time (years)
low NfL
high NfL
mid NfL
AD stage
I
II
III

SFigure 5

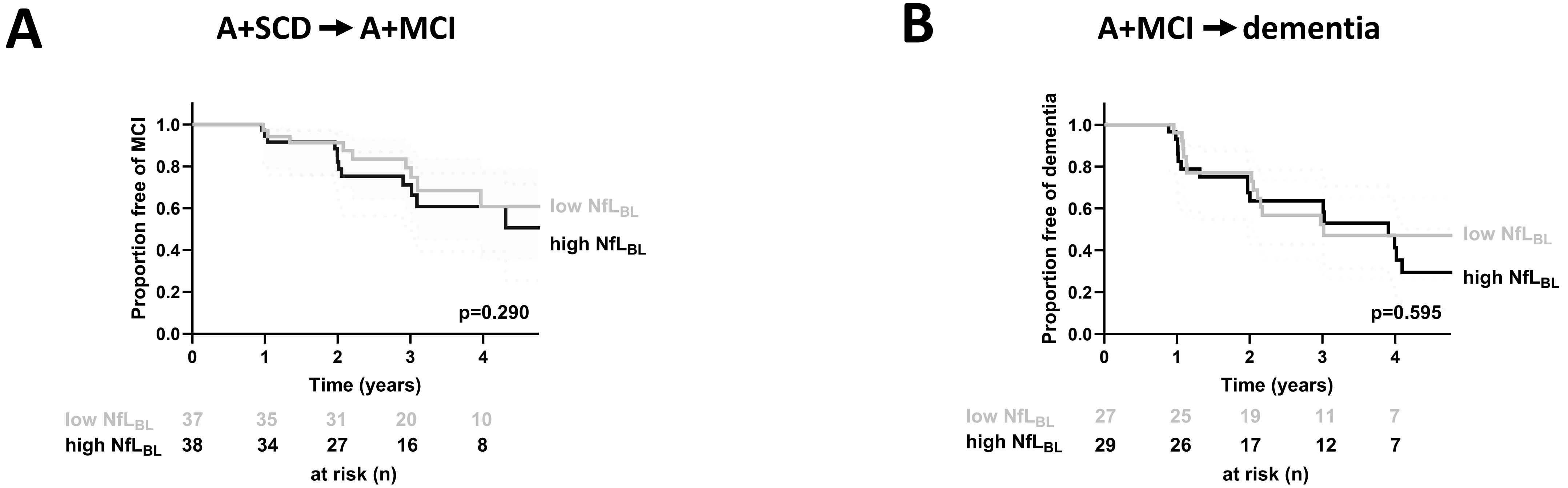
A
A+SCD → A+MCI
Proportion free of MCI
1.0
0.8
0.6
0.4
0.2
0.0
0
1
2
3
4
Time (years)
low NfL_BL
high NfL_BL
p=0.290
low NfL_BL 37 35 31 20 10
high NfL_BL 38 34 27 16 8
at risk (n)
B
A+MCI → dementia
Proportion free of dementia
1.0
0.8
0.6
0.4
0.2
0.0
0
1
2
3
4
Time (years)
low NfL_BL
high NfL_BL
p=0.595
low NfL_BL 27 25 19 11 7
high NfL_BL 29 26 17 12 7
at risk (n)
